# Screening for CKD in undiagnosed populations with diabetes and hypertension: lessons from the SEARCH study

**DOI:** 10.1590/2175-8239-JBN-2025-0231en

**Published:** 2026-01-26

**Authors:** Thyago Proença de Moraes, Ana Flavia Moura, Andrei C. Sposito, Flavia Paiva Proença Lobo Lopes, André Ricardo de Araújo Quadros, Victor Gadelha, Freddy Goldberg Eliaschewitz, Marcello C. Bertoluci, Farid Samaan, Cinthia Montenegro, Tamisa Rego, Roberto Pecoits-Filho

**Affiliations:** 1 Pontifícia Universidade Católica do Paraná, Curitiba, PA, Brazil.; 2 Escola Bahiana de Medicina e Saúde Pública, Salvador, BA, Brazil.; 3 Universidade Estadual de Campinas, Laboratório de Aterosclerose e Biologia Vascular, Campinas, SP, Brazil.; 4 Dasa, São Paulo, SP, Brazil.; 5 Universidade Federal do Rio de Janeiro, Rio de Janeiro, RJ, Brazil.; 6 Centro de Pesquisas Clínicas, São Paulo, SP, Brazil.; 7 Universidade Federal do Rio Grande do Sul, Porto Alegre, RS, Brazil.; 8 Secretaria de Estado da Saúde de São Paulo, SP, Brazil.; 9 Instituto Dante Pazzanese de Cardiologia, São Paulo, SP, Brazil.; 10 AstraZeneca, São Paulo, SP, Brazil.; 11 Arbor Research Collaborative for Health, Ann Arbor, MI, United States.

**Keywords:** Chronic Kidney Disease, Screening Campaign, Albuminuria, KDIGO

## Abstract

**Introduction:**

Despite advances in chronic kidney disease (CKD) management, early diagnosis and risk stratification remain major challenges. This study analyzed national data derived from a Brazilian CKD screening campaign, targeting high-risk individuals unaware of their condition, to estimate CKD prevalence and evaluate longitudinal trends in estimated glomerular filtration rate (eGFR).

**Methods:**

This cross-sectional, observational study evaluated adults with diabetes mellitus (DM) and/or arterial hypertension, previously undiagnosed with CKD, who participated in a CKD screening campaign. Demographic, clinical, and laboratory data—including serum creatinine and albumin-to-creatinine ratio (ACR)—were collected. CKD staging followed Kidney Disease: Improving Global Outcomes (KDIGO) criteria, and eGFR was calculated using the CKD-EPI 2021 equation. Mixed effect models were used to assess eGFR trajectories and logistic regression was used to examine CKD risk factors.

**Results:**

Among 14,239 high-risk participants (mean age 54 years; 84% hypertensive; 37% diabetic), CKD was newly identified in 19.6%, with 11.3% showing eGFR <60 mL/min and 11.1% ACR >30 mg/g. Despite abnormal ACR results, testing remained uncommon: <5% prior to the campaign and <7% afterward. eGFR decline was steeper among diabetics (+40%), hypertensive individuals (+17%), those >65 years of age (+17%), and individuals with body mass index (BMI) >25 kg/m^2^ (+60%). Logistic regression models of risk projected ~169,000 kidney failure cases in 2 years and ~500,000 in 5 years, excluding competing mortality risk.

**Conclusion:**

A high prevalence of previously undiagnosed CKD was found in this high-risk population. Despite universal access to testing in Brazil, albuminuria remains markedly underused, representing a missed opportunity for early detection and implementation of nephroprotective interventions.

## Introduction

Over the past decade, a paradigm shift has occurred in the management of chronic kidney disease (CKD). Guideline-directed medical therapy (GDMT), once limited to pharmacological blockade of the renin-angiotensin-aldosterone system^1^, have expanded to include sodium-glucose cotransporter (SGLT2) inhibitors^2,3,4^, nonsteroidal mineralocorticoid receptor antagonists^5^, and glucagon-like peptide-1 (GLP-1) receptor agonists^6^. These therapies act complementarily, with the potential to slow the annual decline in estimated glomerular filtration rate (eGFR) to levels close to those expected during normal aging^7^.

Despite significant therapeutic advances, early and accurate diagnosis and appropriate risk stratification remain major barriers to reducing CKD-related complications, including progression to advanced stages and increased cardiovascular risk^8^. We have previously described that a significant number of patients with moderate to severe kidney dysfunction based on laboratory values were not coded as patients with CKD in electronic health records in multiple countries^9^. Interestingly, the documentation of CKD in medical records was associated with improved guideline-recommended management and a slower decline in eGFR. These findings highlight that early CKD diagnosis may offer a valuable opportunity to improve patient outcomes^10^.

Recently, a systematic review of CKD screening studies concluded that the strategy is cost-effective in populations at high risk of CKD such as those with DM and arterial hypertension^11^. In Brazil, a population-based study estimated CKD prevalence at approximately 9% among adults^12^, but its prevalence in high risk groups is unknown. The high prevalence of CKD, coupled with its potentially devastating impact on patient quality of life and healthcare resource utilization if not diagnosed in a timely manner and properly managed, has prompted the proposal of screening campaigns targeting high-risk populations as a potential solution.

Guided by this rationale, we analyzed data from a national CKD screening campaign targeting individuals with DM and arterial hypertension who were previously unaware of the diagnosis of CKD, and retrieved information from laboratory records to allow an analysis of patterns of eGFR slopes one year before and one year after the campaign. Our objective was to estimate CKD prevalence and risk stratification cross-sectionally and to evaluate eGFR trajectories over time through retrospective longitudinal analysis.

## Methods

This was a Brazilian cross-sectional, observational, and analytical epidemiological study, with data obtained from the DASA database, concerning patients who participated in the “*Early Diagnosis Campaign for Chronic Kidney Disease*”. The campaign was performed in high-risk patients from February to December 2022. It was sponsored by AstraZeneca and conducted by diagnostic units of a large clinical laboratory network in Brazil - Diagnósticos da América (DASA). In addition, an exploratory retrospective analysis was conducted to evaluate longitudinal changes in eGFR slope using historical laboratory data available in the database. The study was approved by the Research Ethics Committee of Hospital 9 de Julho, and all patients signed the informed consent form. The approval number was 7.189.405.

The campaign recruited patients with DM and/or arterial hypertension (self-report) and no history of CKD. Participants completed a questionnaire on demographic (age and sex) and clinical characteristics (cardiovascular comorbidities and family history). Serum creatinine concentration was measured using the Jaffé method (confirmatory repeat tests were not performed), and a single urine sample was collected to determine the albumin to creatinine ratio (UACR) using the Microalbuminuria Turbiquest Plus kit.

All participants received a report containing a specific note to raise awareness of CKD, explaining their CKD stage according to the KDIGO definition and classification^7^. The eGFR was estimated by the CKD-EPI 2021 equation7. Additionally, laboratory data was retrieved from charts, including serum creatinine, UACR, hemoglobin A1c (HbA1c), and cholesterol measured as part of the clinical care routine 2 years before and 2 years after the campaign period.

### Statistical Analysis

Continuous variables were presented as mean with standard deviation or as median with interquartile range, depending on the variable’s distribution. Categorical variables were reported as percentages along with their corresponding absolute counts. To calculate the eGFR slopes we used a mixed effect regression model, and each patient could contribute with up to 3 eGFRs over a period of 2 years before or after the campaign. Finally, to estimate the risk of CKD at the campaign based on clinical and demographic characteristics we used logistic regression. P value <0.05 was considered significant. All analyses were performed using the STATA 18^®^ software.

## Results

The cohort consisted of 14,239 individuals aged 18 years or older, with a mean age of 54 ± 15 years. Two-thirds were female, 37% self-reported a diagnosis of DM, and 84% self-reported having arterial hypertension. Approximately 20% reported the presence of both comorbidities. Table 1 summarizes the clinical, demographic, and laboratory characteristics of the population.

**Table 1:** Clinical and demographic characteristics

Variable	Result
Age (years)	53.7 ± 14.8
***Inclusion Criteria***		
Diabetes	5,237 (36.8%)
Hypertension	11,870 (83.4%)
Diabetes + Hypertension	2,868 (20.1%)
***Comorbidities***		
Stroke	296 (2.1%)
Heart failure	152 (1.1%)
Acute myocardial infarction	82 (0.6%)
Angina pectoris	309 (2.2%)
Others	441 (3.1%)
Family history of CKD	145 (1.0%)
***Gender***		
Male	4,817 (33.8%)
Female	9,422 (66.2%)
***UACR(mg/g)*** (n = 14,239)	4.8 (2.2-11.7)
***eGFR*** (CKDEPI) (n = 14,239)	83.7 ± 19.8
Total Cholesterol (mg/dL) (n = 8.679)	184.9 ± 41.7
Cholesterol HDL (mg/dL) (n = 8.617)	52.4 ± 15.0
Cholesterol LDL (mg/dL) (n = 8.572)	109.2 ± 35.8
Cholesterol VLDL (mg/dL) (n = 8.560)	23.1 ± 10.2
Triglycerides (mg/dL) (n = 8.679)	127.4 ± 82.0
**HbA1c (%)**		
Diabetes (n = 3,262)	6.6 ± 1.5
Without diabetes (n = 4,996)	5.7 ± 0.7
**KDIGO stage**	N = 14.239
A1	12,627 (88.7%)
A2	1,327 (9.3%)
A3	285 (2.0%)
G1	5,486 (38.5%)
G2	7,133 (50.2%)
G3a	1,183 (8.3%)
G3b	333 (2.3%)
G4	84 (0.6%)
G5	20 (0.1%)

**Figure 1:**
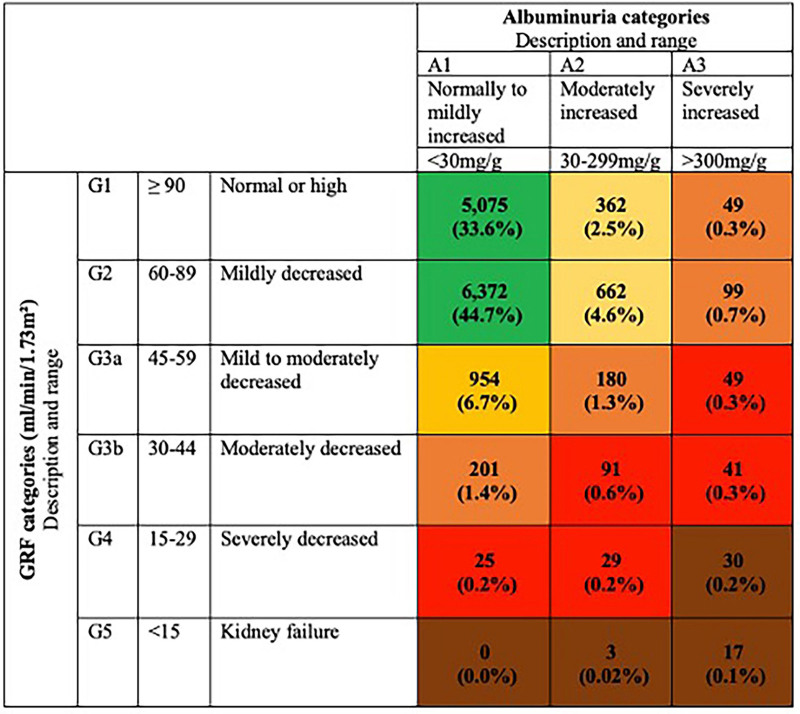
Stage stratification in the campaign.

Using the 2024 KDIGO classification, nearly 19.6% (n = 2,792) of the cohort had CKD, all without prior knowledge of the condition (Figure 1). Among them, 11.3% had a eGFR below 60 mL/min (n = 1,620), and 11.1% (n = 1,612) had albuminuria greater than 30 mg/g. The simultaneous presence of both diagnostic criteria was observed in only 3% (n = 440) of the population.

We also evaluated the risk factors for an eGFR below 60 mL/min or a UACR above 30 mg/g at the time of the campaign. Having DM, being male, being over 65 years old, and having a BMI above 25 kg/m^2^ were risk factors for both reduced eGFR and elevated albuminuria. Patients with arterial hypertension were associated with reduced eGFR but not with increased albuminuria (Table 2).

**Table 2:** Characteristics associated with a high risk of CKD at the campaign

Group	Odds ratio for eGFR < 60 mL/min	Odds ratio for UACR > 30 mg/g
*Diabetes* (Yes)	1.54 (1.39–1.71)	1.96 (1.77-2.18)
*Hypertension* (Yes)	1.44 (1.23–1.68)	0.94 (0.82–1.08)
*Diabetes + hypertension*	2.22 (1.99–2.49)	2.28 (2.04–2.55)
*Sex* (Male)	1.21 (1.08–1.35)	3.64 (3.21–4.12)
*Age* (>65 years)	8.00 (7.14–8.91)	2.31 (2.07–2.57)
*BMI* (> 25 kg/m2)	1.40 (0.95–2.06)	1.78 (1.16–2.73)

Abbreviation – BMI: Body mass index.

We obtained HbA1c results from 8,258 patients near the time of the campaign. Regarding glycemic control among patients with DM, the mean HbA1c levels was 6.6 ± 1.5%, and 35% (n = 1,148) had an HbA1c above 6.5%. Among non-diabetic individuals, 7% had HbA1c levels consistent with DM, and 35% had levels indicative of impaired glucose tolerance (values between 5.7% and 6.4%).

The request for albuminuria tests before or after the campaign was extremely low. Less than 5% of participants who had their creatinine measured also had an UACR performed at the same laboratory within the previous 2 years. In the current study, despite a significant absolute increase of 33% (from 628 to 939) in requests for UACR following the campaign, the percentage of requests for UACR remained low, at just under 7% in the general population and 10% in the subgroup with an eGFR below 60 mL/min during the campaign. Even in the subgroup where UACR exceeded 30 mg/g during the campaign, only 8.8% had a UACR test repeated alongside other creatinine measurements within the 2 years following the event.

### eGFR Slope in the Two Years Preceding and Succeeding the Screening Campaign

Among the campaign population, 6,808 patients had at least one creatinine measurement taken within the two years preceding the study, all performed in the clinical analysis network used for this work. The demographic characteristics of this group were largely similar to the total population. The mean age was 55 ± 15 years, with 39.4% having DM and 82.3% arterial hypertension.

**Figure 2:**
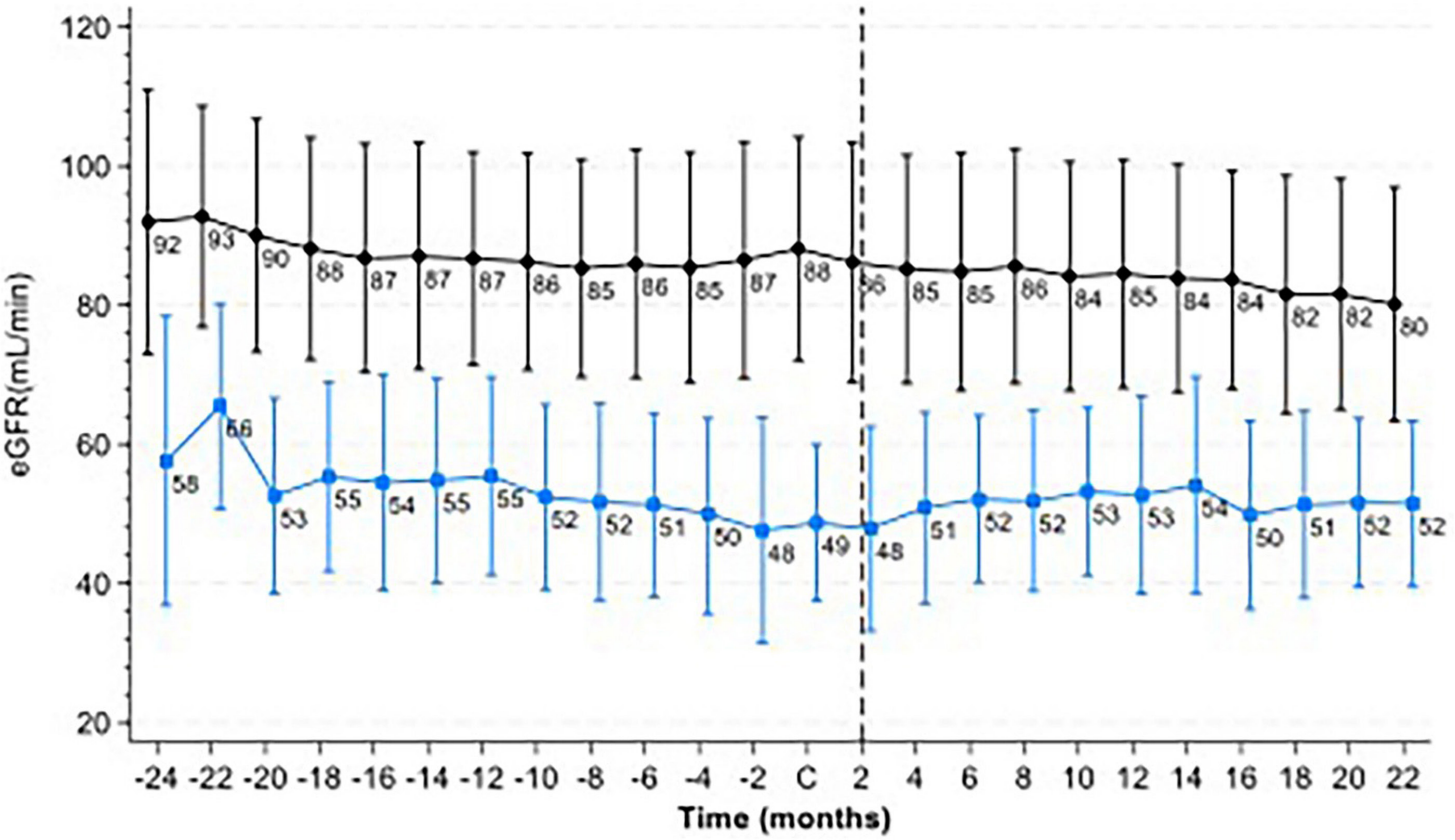
Mean eGFR before and after the campaign.

The overall eGFR trajectory revealed an annual decline (slope) of 1.06 mL/min (95% CI: 0.84–1.29). However, this decline varied significantly across specific subgroups (Figure 2). Among patients with a eGFR below 60 mL/min during the campaign, the annual slope decrease was 5.2 mL/min (95% CI: 4.60–5.80), compared to 0.48 mL/min (95% CI: 0.25–0.72) in those with a eGFR of 60 mL/min or higher. Key subgroup differences included (a) diabetic patients had an annual decline nearly 40% greater than non-diabetics; (b) hypertensive patients had a 17% greater decline compared to non-hypertensives; (c) patients over 65 years also had a 17% faster decline than younger individuals; and (d) people with a BMI > 25 kg/m^2^ had a decline rate 60% higher than those with a BMI ≤ 25 kg/m^2^. Table 3 provides a detailed summary of the annual eGFR decline for each subgroup.

Note – Black (eGFR>=60mL/min at the campaign); Blue (eGFR<60mL/min at the campaign).

**Table 3:** Risk of kidney failure at 2 and 5 years after the diagnosis

	Two-year risk of event *(median (P10-P90))*	Five-year risk of progression *(median (P10-P90))*
Overall	0.2% (0–4)	0.8% (0–16)
*Diabetes*		
Yes	0.3% (0–7)	1% (0–25)
No	0.2% (0–3)	0.6% (0–12)
*Hypertension*		
Yes	0.2% (0–4)	0.7% (0–12)
No	0.2% (0–3)	0.8% (0–17)
*Diabetes &* *Hypertension*	0.4% (0–11)	1.6% (0–42)
*Sex*		
Men	0.3% (0–6)	1.0% (0–24)
Women	0.2% (0–3)	0.6% (0–12)
*Age*		
>65 year	0.9% (0–17)	3.0% (3–99)
65 or less	0.1% (0–2)	0.5% (0–7)
*Body mass index*		
< 25 kg/m2	0.1% (0–2)	0.5% (0–9)
25 to 29.9 kg/m2	0.2% (0–4)	0.8% (0–15)

### Renal Function Behavior in the Two Years Following the Campaign

In the two years after the campaign, 6,970 patients underwent at least one additional creatinine measurement. The demographic characteristics of this subgroup remained consistent with the overall population, with a mean age of 55 ± 15 years and 39% diagnosed with DM.

Post-campaign eGFR trends showed distinct patterns based on baseline kidney function. Among patients with a eGFR above 60 mL/min at the time of the campaign, the annual decline was 2.7 mL/min (95% CI: 2.30–3.05). Conversely, those with an eGFR below 60 mL/min exhibited remarkable stability over the follow-up period, with a negligible annual change of -0.2 mL/min (95% CI: -0.88 to 0.84).

### Risk of Kidney Failure

We also used the Kidney Failure Risk Equation to estimate the probability of kidney failure within 2 and 5 years after diagnosis. The median risk for patients meeting CKD diagnostic criteria, whether based on eGFR or UACR > 30 mg/g, was 4.1% at 2 years and 15.8% at 5 years. In the population of 1,630 patients for whom we estimated the risk (all individuals with CKD in the cohort), it was projected that 14 patients will progress to kidney failure within 2 years and 41 within 5 years (Table 3).

## Discussion

Population-based studies estimating CKD prevalence remain limited in low- and middle-income countries. In a high-risk population participating in a national screening campaign, we found that 1 in 5 patients with DM and/or arterial hypertension met the KDIGO criteria for CKD. Our findings were similar to data reported in a large NHANES cohort from 2007 to 2018, where 17.5% of adults at risk for CKD were identified with albuminuria above 30 mg/g^13^. While methodologies differ, this similarity reinforces the burden of undetected CKD in high-risk populations.

According to global estimates, nearly 10% of the world’s population has CKD across its various stages. In comparison to a previous Brazilian population-based study, the ELSA study, the prevalence reported here for diabetic and/or hypertensive patients was more than double in the general adult population^12^. In Brazil, the Brazilian Society of Diabetes reports that there are around 20 million adults with DM, and 60% of them will develop CKD at some point. Our estimates, using a validated equation, indicate the alarming projection of approximately 169,000 new cases of kidney failure in diabetic and hypertensive patients in the next 2 years and 500,000 within 5 years. These are alarming data, consistent with what we have observed over the years in the Brazilian Society of Nephrology’s census. Over the past 15 years, the number of patients on dialysis has doubled, rising from 71,000 in 2006 to 154,000 in 2022^14^. Considering the innovations that have enabled better management of factors contributing to cardiovascular mortality, the estimated number of underdiagnosed patients, and the findings of this study, the outlook for public health could worsen if intervention measures are not implemented. It is important to mention that this estimate does not include cases of fatal cardiovascular events before kidney failure.

Another important finding of our study, in addition to the high prevalence of CKD, was the extremely low rate of albuminuria testing both before and after the campaign. Albuminuria, a key measure for both diagnosing and risk stratifying patients with the disease, is often overlooked in clinical practice, not only in developing countries but also in developed ones. We anticipated that following the campaign, there would be a significant increase in the use of the UACR, particularly in the population where we identified A2 or A3 albuminuria according to KDIGO criteria. To our surprise, fewer than 9% of patients with A2 or A3 albuminuria identified during the campaign had a new ACR measured when a creatinine test was requested.

The current study confirm our previous results published with the Check CKD study, based on more than 4.5 million creatinine tests requested between 2018 and 2021^8^. In Check CKD, the numbers of albuminuria tests alongside creatinine were higher, but this included patients who were already being monitored and aware of their CKD diagnosis. Other studies have shown a testing rate of 29% in France and 21% in the United States in a subgroup of patients where albuminuria should have been measured in all cases^15,16^. In the study by Qiao et al.^17^, among a subgroup of 35,000 patients without RAAS inhibitors in their prescriptions, individuals with A2 and A3 albuminuria saw an increase in the prescription of this drug class by 37% and 43%, respectively. The study reinforces that the measurement of albuminuria has been associated with increased prescription of RAASi^17^. Identifying CKD by eGFR levels complemented with albuminuria prompts clinicians to initiate or intensify nephroprotective therapies, improving adherence to guideline-based care. In our study, however, fewer than 10% of individuals who had a serum creatinine test also underwent albuminuria testing, even after receiving their campaign results.

CKD patient awareness campaigns, as the one presented in this study, are essential for improving early detection, timely interventions, proper referral to nephrologists, and effective disease management. Beyond individual benefits, patient awareness may play a pivotal role in shared decision-making and fostering better communication between patients and healthcare providers. These actions could slow disease progression, reduce complications, enhance overall quality of life, and reduce the economic burden of CKD.

**Figure 3:**
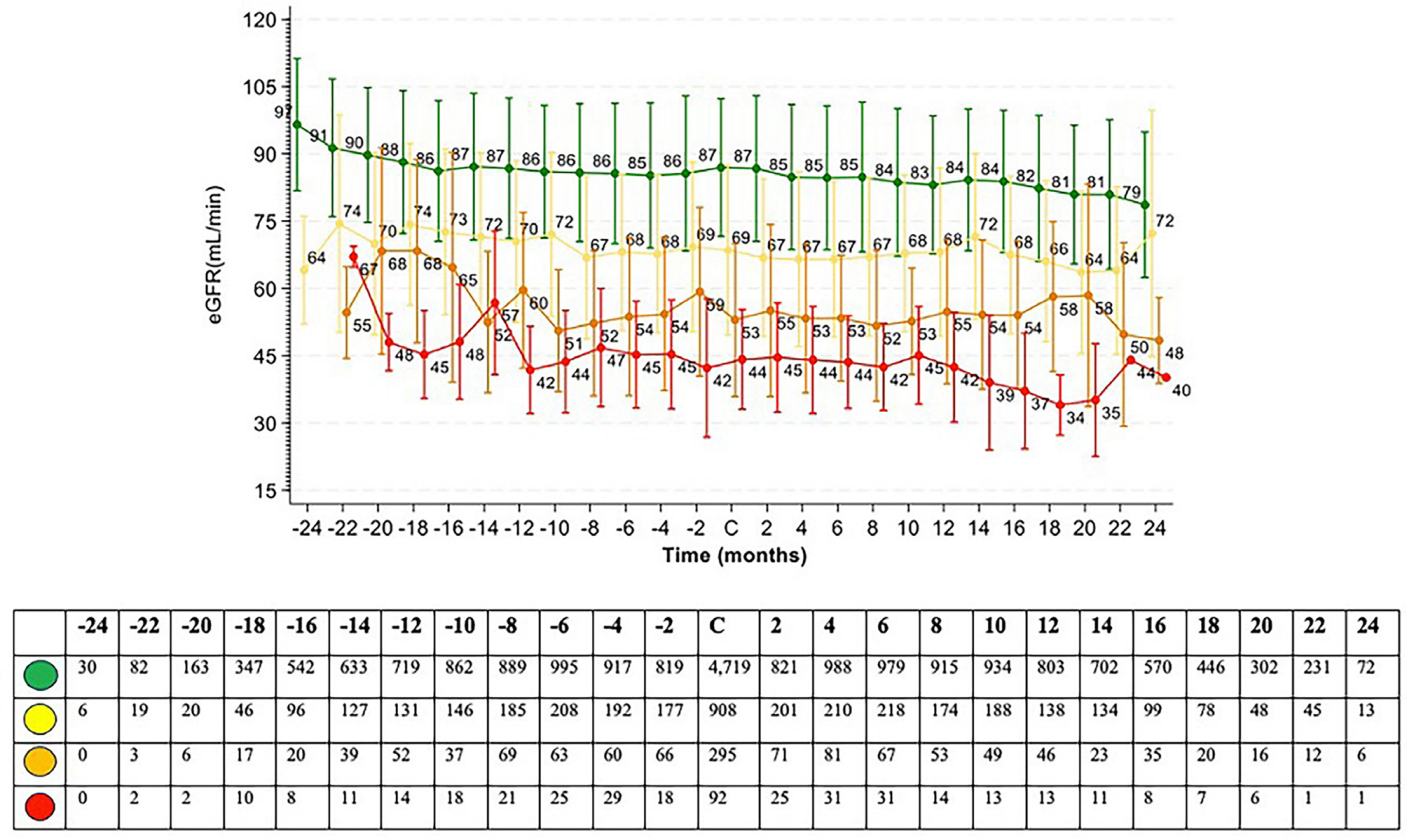
Mean eGFR stratified by KDIGO risk of progression.

**Figure 4:**
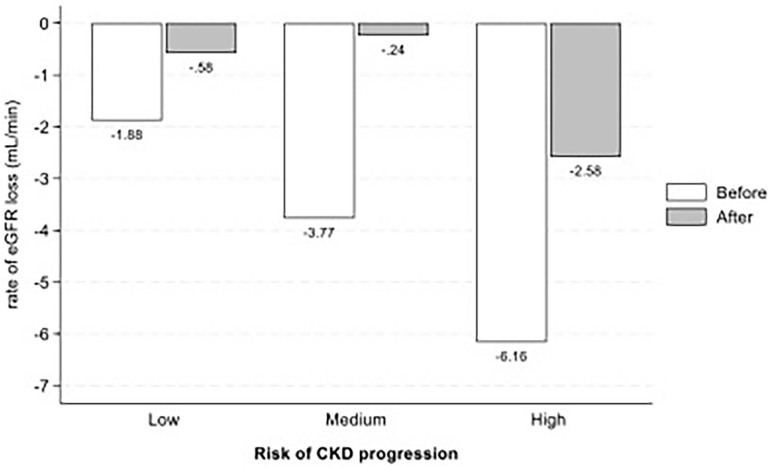
Annual eGFR slope before and after the campaign.

The eGFR slope provides a robust and sensitive measure of kidney function decline over time, offering significant advantages for both research and clinical applications. In observational studies, the eGFR slope allows for the assessment of disease progression and the identification of risk factors in real-world settings. Meanwhile, in RCTs, it serves as a surrogate endpoint that can detect treatment effects earlier and more precisely than traditional endpoints, such as the progression to end-stage kidney disease or mortality. For this reason, we also explored these data during the periods preceding and following the campaign. We observed that patients with some degree of CKD, whether albuminuria or reduced eGFR, experienced a faster progression in the year preceding the campaign than would typically be expected with normal aging. The annual decline of approximately 1 mL/min per year in the group without kidney disease, classified as green by KDIGO, underscores the quality of the data presented, despite the absence of critical information regarding the management of risk factors for kidney disease progression (Figure 3). An interesting finding, despite the inherent limitations of our study design, was the reduction in the rate of eGFR decline across the three subgroups with chronic kidney disease (Figure 4). Although only a small percentage of patients had albuminuria measured over the two years following diagnosis, this may indirectly highlight one of the potential clinical benefits of screening campaigns. Patients with eGFR <60 mL/min are more likely to be recognized as having CKD and thus receive appropriate treatment according to GDMT. In contrast, those with risk factors but preserved eGFR ≥60 mL/min are less likely to be managed as high-risk, which may explain the persistent risk observed in this subgroup. However, our study was not designed to capture this mechanism, and this remains a speculative interpretation.

Notes – Estimated annual slope preceding the campaign: No CKD (Green) –1.39 (–0.90; –1.88); Low risk (Yellow): –1.88 (–0.77; –2.99); Medium risk (Orange): –3.77 (–3.37; –8.96); High risk (Red): –6.16(–8.96; –3.37). Estimated annual slope succeeding the campaign: No CKD (Green) –2.79 (–2.52;-3.06); Low risk (Yellow): –0.58 (0.04; –1.19); Medium risk (Orange): –0.24 (1.06; –1.54); High risk (Red): –2.58(-0.69; –4.47).

Patients who did not meet the CKD criteria during the campaign showed a slower decline in eGFR compared to those who met the criteria (data not shown). An additional exploratory analysis of key subgroups revealed that older adults, men, individuals with DM or obesity experienced a more rapid decline in eGFR.

Finally, within our cohort, elderly patients, those with DM, overweight or obesity, and males were more likely to present both reduced eGFR and albuminuria during the screening campaign. Conversely, hypertensive patients showed a higher risk for reduced eGFR but not for albuminuria. However, it is important to note that our study included only patients with at least one comorbidity, as this was an inclusion criterion. Therefore, it cannot be concluded whether the risk of albuminuria would have been elevated if the comparator had been non-hypertensive, non-diabetic individuals, since the reference was necessarily individuals with DM. Nonetheless, the prevalence of hypertensive patients with an ACR > 30 mg/g in our study was four times lower than that reported in a large Portuguese cohort study^13^.

Our study has some limitations. It was conducted primarily in southeastern cities, which may limit generalizability. Participation was voluntary, possibly favoring individuals with better healthcare access than the general population, potentially influencing prevalence estimates and testing patterns. Data may be incomplete for participants who performed tests outside the study’s laboratory network. Although testing followed standardized procedures, multiple sites were involved. Only a single eGFR and ACR measurement was used, and confirmatory testing within 90 days was rarely feasible. The use of the Jaffe method for creatinine measurement is another limitation, as this assay is subject to analytical interferences (e.g., glucose, ketones, bilirubin, certain drugs), which may slightly bias results despite standardized calibration. Finally, the study did not assess whether early CKD diagnosis led to improved outcomes or treatment adherence.

Despite these limitations, campaigns like the one reported in this study are essential for improving early CKD detection, raising public awareness, and encouraging high-risk individuals to seek medical care. In Brazil, both serum creatinine and UACR tests (as well as RAASi and SGLT2i) are universally available through the public healthcare system, with no formal barriers to their request. Nevertheless, underdiagnosis and incomplete risk stratification, and underutilization of GDMT persists. Such initiatives play a crucial role in developing targeted strategies to prevent CKD progression and reduce its long-term health and economic burden.

In conclusion, we described a high prevalence of CKD among individuals with DM and arterial hypertension without history of CKD, and significant challenges persist in complementing eGFR with albuminuria testing—the earliest biomarker for CKD detection—in clinical practice. The interesting trends in eGFR slope before and after the campaign (which included providing participants with their CKD screening results) suggest that identifying and risk-stratifying patients, along with raising CKD awareness, may influence outcomes, potentially through changes in practice pattern and improved management. This observation warrants further investigation, but supports the value of CKD screening in high-risk populations.

## Data Availability

The datasets generated and/or analyzed during the current study are available from the corresponding author upon reasonable request.
