# Supplementary material for: Screening for CKD in undiagnosed populations with diabetes and hypertension: lessons from the SEARCH study
Source: J Bras Nefrol. 2026 Jan 26;48(2):e20250231. doi: 10.1590/2175-8239-JBN-2025-0231en (PMC12854714; doi:10.1590/2175-8239-JBN-2025-0231en)
Supplement: Translation

# Translation: Rastreamento de DRC em população com diabetes e hipertensão, mas sem diagnóstico conhecido de doença renal: lições extraídas do estudo SEARCH

**DOI:** 10.1590/2175-8239-JBN-2025-0231pt

**Authors:** Thyago Proença de Moraes, Ana Flavia Moura, Andrei C. Sposito, Flavia Paiva Proença Lobo Lopes, André Ricardo de Araújo Quadros, Victor Gadelha, Freddy Goldberg Eliaschewitz, Marcello C. Bertoluci, Farid Samaan, Cinthia Montenegro, Tamisa Rego, Roberto Pecoits-Filho

**Affiliations:** 1 Pontifícia Universidade Católica do Paraná, Curitiba, PA, Brasil.; 2 Escola Bahiana de Medicina e Saúde Pública, Salvador, BA, Brasil.; 3 Universidade Estadual de Campinas, Laboratório de Aterosclerose e Biologia Vascular, Campinas, SP, Brasil.; 4 Dasa, São Paulo, SP, Brasil.; 5 Universidade Federal do Rio de Janeiro, Rio de Janeiro, RJ, Brasil.; 6 Centro de Pesquisas Clínicas, São Paulo, SP, Brasil.; 7 Universidade Federal do Rio Grande do Sul, Porto Alegre, RS, Brasil.; 8 Secretaria de Estado da Saúde de São Paulo, SP, Brasil.; 9 Instituto Dante Pazzanese de Cardiologia, São Paulo, SP, Brasil.; 10 AstraZeneca, São Paulo, SP, Brasil.; 11 Arbor Research Collaborative for Health, Ann Arbor, MI, United States.

**Keywords:** Doença Renal Crônica, Campanha de Rastreamento, Albuminúria, KDIGO

## Abstract

**Introdução:**

Apesar dos avanços no manejo da doença renal crônica (DRC), o diagnóstico precoce e a estratificação de risco permanecem grandes desafios. Este estudo analisou dados nacionais derivados de uma campanha brasileira de rastreamento da DRC, voltada para indivíduos de alto risco que desconheciam sua condição, visando estimar a prevalência da doença e avaliar tendências longitudinais na taxa de filtração glomerular estimada (TFGe).

**Métodos:**

Estudo transversal observacional avaliando adultos com diabetes mellitus (DM) e/ou hipertensão arterial, sem diagnóstico prévio de DRC, que participaram de campanha de rastreamento da doença. Coletaram-se dados demográficos, clínicos e laboratoriais, incluindo creatinina sérica e relação albumina/creatinina (RAC). O estadiamento da DRC seguiu os critérios do *Kidney Disease: Improving Global Outcomes* (KDIGO) e calculou-se a TFGe pela equação CKD-EPI 2021. Modelos de efeitos mistos foram utilizados para avaliar as trajetórias da TFGe e regressão logística para examinar fatores de risco para DRC.

**Resultados:**

Entre 14.239 participantes de alto risco (idade média 54 anos; 84% hipertensos; 37% diabéticos), a DRC foi identificada pela primeira vez em 19,6%, com 11,3% apresentando TFGe <60 mL/min e 11,1% RAC >30 mg/g. Apesar dos resultados anormais de RAC, os testes permaneceram incomuns: <5% antes da campanha e <7% depois. O declínio da TFGe foi mais acentuado entre diabéticos (+40%), indivíduos hipertensos (+17%), aqueles com idade >65 anos (+17%) e indivíduos com índice de massa corporal (IMC) >25 kg/m^2^ (+60%). Modelos de regressão logística de risco projetaram ~169.000 casos de insuficiência renal em 2 anos e ~500.000 em 5 anos, excluindo o risco competitivo de mortalidade.

**Conclusão:**

Identificamos uma prevalência elevada de DRC previamente não diagnosticada nesta população de alto risco. Apesar do acesso universal a exames no Brasil, a albuminúria segue sendo significativamente subutilizada, representando uma oportunidade perdida para detecção precoce e implementação de intervenções nefroprotetoras.

## Introdução

Ao longo da última década, houve uma mudança de paradigma no manejo da doença renal crônica (DRC). A terapia médica orientada por diretrizes (GDMT, do inglês *guideline-directed medical therapy*), antes limitada ao bloqueio farmacológico do sistema renina-angiotensina-aldosterona^1^, expandiu-se para incluir inibidores do cotransportador sódio-glicose 2 (SGLT2)^2-4^, antagonistas não esteroidais do receptor mineralocorticóide^5^ e agonistas do receptor do peptídeo semelhante ao glucagon-1 (GLP-1)^6^. Essas terapias atuam de forma complementar, com o potencial de retardar o declínio anual da taxa de filtração glomerular estimada (TFGe) para níveis próximos aos esperados durante o envelhecimento normal^7^.

Apesar dos avanços terapêuticos significativos, o diagnóstico precoce e preciso, bem como a estratificação adequada do risco, permanecem como as principais barreiras para a redução de complicações relacionadas à DRC, incluindo a progressão para estágios avançados e o aumento do risco cardiovascular^8^. Anteriormente, descrevemos que um número significativo de pacientes com disfunção renal moderada a grave, com base em valores laboratoriais, não foi codificado como portador de DRC nos prontuários eletrônicos de saúde em diversos países^9^. Curiosamente, a documentação da DRC nos prontuários médicos esteve associada a uma melhora no manejo recomendado pelas diretrizes e a um declínio mais lento na TFGe. Esses achados ressaltam que o diagnóstico precoce da doença pode oferecer uma oportunidade valiosa para aprimorar os desfechos dos pacientes^10^.

Recentemente, uma revisão sistemática de estudos de rastreamento da DRC concluiu que a estratégia é custo-efetiva em populações de alto risco para a doença, como aquelas com DM e hipertensão arterial^11^. No Brasil, um estudo de base populacional estimou a prevalência de DRC em aproximadamente 9% entre adultos^12^, mas a frequência dessa condição em grupos de alto risco permanece desconhecida. A elevada prevalência da doença, aliada ao seu potencial impacto devastador na qualidade de vida dos pacientes e na utilização de recursos de saúde – caso não seja diagnosticada oportunamente e submetida a um manejo adequado – motivou a proposta de campanhas de rastreamento direcionadas a populações de alto risco como uma possível solução.

Guiados por essa lógica, analisamos dados de uma campanha nacional de rastreamento de DRC voltada para indivíduos com DM e hipertensão arterial que desconheciam anteriormente o diagnóstico da doença, e recuperamos informações de registros laboratoriais para permitir uma análise dos padrões das inclinações da TFGe no ano anterior e no ano posterior à campanha. Nosso objetivo foi estimar, de forma transversal, a prevalência da DRC e a estratificação de risco, bem como avaliar as trajetórias da TFGe ao longo do tempo por meio de uma análise longitudinal retrospectiva.

## Métodos

Este foi um estudo epidemiológico brasileiro transversal, observacional e analítico, com dados obtidos a partir do banco de dados da Diagnósticos da América (DASA), referente a pacientes que participaram da “Campanha de Diagnóstico Precoce da Doença Renal Crônica”. A campanha foi realizada em pacientes de alto risco, de fevereiro a dezembro de 2022. Foi patrocinada pela AstraZeneca e conduzida por unidades diagnósticas de uma ampla rede de laboratórios clínicos no Brasil – a DASA. Além disso, foi realizada uma análise retrospectiva exploratória com o objetivo de avaliar as alterações longitudinais na inclinação da TFGe utilizando dados laboratoriais históricos disponíveis na base de dados. O estudo foi aprovado pelo Comitê de Ética em Pesquisa do Hospital 9 de Julho, e todos os pacientes assinaram o termo de consentimento livre e esclarecido. O número da aprovação foi 7.189.405.

A campanha recrutou pacientes com DM e/ou hipertensão arterial (autorreferidos) e sem histórico de DRC. Os participantes preencheram um questionário sobre características demográficas (idade e sexo) e clínicas (comorbidades cardiovasculares e histórico familiar). A concentração sérica de creatinina foi mensurada pelo método de *Jaffé* (não foram realizados testes confirmatórios repetidos) e uma única amostra de urina foi coletada para determinação da relação albumina/creatinina (RACU) utilizando o kit Microalbuminúria Turbiquest Plus.

Todos os participantes receberam um laudo contendo uma nota específica de conscientização sobre a DRC, explicando o estágio em que se encontravam de acordo com a definição e a classificação do KDIGO^7^. A TFGe foi estimada pela equação CKD-EPI 2021^7^. Além disso, foram recuperados dados laboratoriais dos prontuários, incluindo creatinina sérica, RACU, hemoglobina glicada (HbA1c) e colesterol, mensurados como parte da rotina de cuidados clínicos dois anos antes e dois anos após o período da campanha.

### Análise Estatística

As variáveis contínuas foram apresentadas como média e desvio padrão ou mediana com intervalo interquartil, dependendo da distribuição da variável. As variáveis categóricas foram relatadas como porcentagens, juntamente com suas contagens absolutas correspondentes. Para calcular as inclinações da TFGe, foi utilizado um modelo de regressão de efeitos mistos, e cada paciente pôde contribuir com até três medidas de TFGe durante um período de dois anos antes ou após a campanha. Por fim, para estimar o risco de DRC no momento da campanha com base nas características clínicas e demográficas, utilizamos regressão logística. Um valor de p <0,05 foi considerado significativo. Todas as análises foram realizadas utilizando o software STATA 18^®^.

## Resultados

A coorte foi composta por 14.239 indivíduos com 18 anos ou mais, com idade média de 54 ± 15 anos. Dois terços eram do sexo feminino, 37% autorreferiram diagnóstico de DM e 84% relataram ter hipertensão arterial. Aproximadamente 20% relataram a presença de ambas as comorbidades. A Tabela 1 resume as características clínicas, demográficas e laboratoriais da população.

**Tabela 1:** Características clínicas e demográficas

Variável	Resultado
Idade (anos)	53,7 ± 14,8
***Critérios de inclusão***	
Diabetes	5.237 (36,8%)
Hipertensão	11.870 (83,4%)
Diabetes + Hipertensão	2.868 (20,1%)
***Comorbidades***	
Acidente vascular cerebral	296 (2,1%)
Insuficiência cardíaca	152 (1,1%)
Infarto agudo do miocárdio	82 (0,6%)
*Angina pectoris*	309 (2,2%)
Outros	441 (3,1%)
Histórico familiar de DRC	145 (1,0%)
***Sexo***	
Masculino	4.817 (33,8%)
Feminino	9.422 (66,2%)
***RACU(mg/g)*** (n = 14.239)	4,8 (2,2–11,7)
***TFGe*** *(CKDEPI)* (n = 14.239)	83,7 ± 19,8
Colesterol total (mg/dL) (n = 8,679)	184,9 ± 41,7
Colesterol HDL (mg/dL) (n = 8,617)	52,4 ± 15,0
Colesterol LDL (mg/dL) (n = 8,572)	109,2 ± 35,8
Colesterol VLDL (mg/dL) (n = 8,560)	23,1 ± 10,2
Triglicérides (mg/dL) (n = 8,679)	127,4 ± 82,0
**HbA1c (%)**	
Diabetes (n = 3.262)	6,6 ± 1,5
Sem diabetes (n = 4.996)	5,7 ± 0,7
**Estágio KDIGO**	N = 14.239
A1	12.627 (88,7%)
A2	1.327 (9,3%)
A3	285 (2,0%)
G1	5.486 (38,5%)
G2	7.133 (50,2%)
G3a	1.183 (8,3%)
G3b	333 (2,3%)
G4	84 (0,6%)
G5	20 (0,1%)

Utilizando a classificação KDIGO 2024, 19,6% (n = 2.792) da coorte apresentou DRC, todos sem conhecimento prévio da condição (Figura 1). Entre esses indivíduos, 11,3% apresentaram TFGe abaixo de 60 mL/min (n = 1.620) e 11,1% (n = 1.612) apresentaram albuminúria superior a 30 mg/g. A presença simultânea de ambos os critérios diagnósticos foi observada em apenas 3% (n = 440) da população.

**Figura 1:** Estratificação por estágio durante a campanha.

Também avaliamos os fatores de risco para uma TFGe inferior a 60 mL/min ou uma RACU superior a 30 mg/g no momento da campanha. A presença de DM, o sexo masculino, a idade superior a 65 anos e um IMC acima de 25 kg/m^2^ foram fatores de risco tanto para redução da TFGe quanto para albuminúria elevada. Pacientes com hipertensão arterial estiveram associados à redução da TFGe, mas não à elevação da albuminúria (Tabela 2).

**Tabela 2:** Características associadas a um alto risco de DRC durante a campanha

Grupo	*Odds ratio* para TFGe < 60 mL/min	*Odds ratio* para RACU > 30 mg/g
*Diabetes* (Sim)	1,54 (1,39–1,71)	1,96 (1,77–2,18)
*Hipertensão* (Sim)	1,44 (1,23–1,68)	0,94 (0,82–1,08)
*Diabetes + hipertensão*	2,22 (1,99–2,49)	2,28 (2,04–2,55)
*Sexo* (Masculino)	1,21 (1,08–1,35)	3,64 (3,21–4,12)
*Idade* (> 65 anos)	8,00 (7,14–8,91)	2,31 (2,07–2,57)
*IMC* (> 25 kg/m2)	1,40 (0,95–2,06)	1,78 (1,16–2,73)

Abreviatura – IMC: Índice de massa corporal.

Obtivemos resultados de HbA1c de 8.258 pacientes de períodos próximos à data da campanha. Em relação ao controle glicêmico entre pacientes com DM, o valor médio de HbA1c foi de 6,6 ± 1,5%, e 35% (n = 1.148) apresentaram HbA1c acima de 6,5%. Entre os indivíduos não diabéticos, 7% apresentaram níveis de HbA1c compatíveis com DM e 35% apresentaram valores indicativos de tolerância à glicose diminuída (valores entre 5,7% e 6,4%).

A solicitação de exames de albuminúria antes ou após a campanha foi extremamente baixa. Menos de 5% dos participantes que tiveram sua creatinina avaliada também realizaram uma análise da RACU no mesmo laboratório nos dois anos anteriores. No presente estudo, apesar de um aumento absoluto significativo de 33% (de 628 para 939) nas solicitações de RACU após a campanha, a porcentagem dessas solicitações permaneceu baixa, pouco menos de 7% na população geral e 10% no subgrupo com TFGe abaixo de 60 mL/min durante a ação. Mesmo no subgrupo em que a RACU excedeu 30 mg/g no período da campanha, apenas 8,8% repetiram o teste de RACU juntamente com outras medições de creatinina nos dois anos subsequentes ao evento.

### Inclinação da TFGe Nos Dois Anos Anteriores e Posteriores à Campanha de Rastreamento

Entre a população da campanha, 6.808 pacientes realizaram pelo menos uma medição de creatinina nos dois anos anteriores ao estudo, todas feitas na rede de análises clínicas utilizada para este trabalho. As características demográficas deste grupo foram amplamente semelhantes às da população total. A idade média foi de 55 ± 15 anos, com 39,4% apresentando DM e 82,3% hipertensão arterial.

A trajetória geral da TFGe revelou um declínio anual (inclinação) de 1,06 mL/min (IC 95%: 0,84–1,29). No entanto, esse declínio variou significativamente entre subgrupos específicos (Figura 2). Entre os pacientes com TFGe inferior a 60 mL/min durante a campanha, a redução anual da inclinação foi de 5,2 mL/min (IC 95%: 4,60–5,80), em comparação com 0,48 mL/min (IC 95%: 0,25–0,72) naqueles com TFGe igual ou superior a 60 mL/min. As principais diferenças entre os subgrupos incluíram: (a) pacientes diabéticos apresentaram um declínio anual quase 40% maior do que os não diabéticos; (b) pacientes hipertensos apresentaram um declínio 17% maior em comparação aos não hipertensos; (c) pacientes com mais de 65 anos também apresentaram um declínio 17% mais rápido do que indivíduos mais jovens; e (d) pessoas com IMC > 25 kg/m^2^ apresentaram uma taxa de declínio 60% superior àquelas com IMC ≤ 25 kg/m^2^. A Tabela 3 fornece um resumo detalhado do declínio anual da TFGe para cada subgrupo.

**Figura 2:** TFGe média antes e após a campanha.

Nota – Preto (TFGe >=60mL/min na campanha); Azul (TFGe <60mL/min na campanha).

**Tabela 3:** Risco de insuficiência renal em 2 e 5 anos após o diagnóstico

	Risco de evento em dois anos *(mediana (P10–P90))*	Risco de progressão em cinco anos *(mediana (P10–P90))*
Total	0,2% (0–4)	0,8% (0–16)
*Diabetes*		
Sim	0,3% (0–7)	1% (0–25)
Não	0,2% (0–3)	0,6% (0–12)
*Hipertensão*		
Sim	0,2% (0–4)	0,7% (0–12)
Não	0,2% (0–3)	0,8% (0–17)
*Diabetes & Hipertensão*	0,4% (0–11)	1,6% (0–42)
*Sexo*		
Masculino	0,3% (0–6)	1,0% (0–24)
Feminino	0,2% (0–3)	0,6% (0–12)
*Idade*		
>65 anos	0,9% (0–17)	3,0% (3–99)
65 ou menos	0,1% (0–2)	0,5% (0–7)
*Índice de massa corporal*		
< 25 kg/m2	0,1% (0–2)	0,5% (0–9)
25 a 29,9 kg/m2	0,2% (0–4)	0,8% (0–15)

### Comportamento da Função Renal nos Dois Anos Seguintes à Campanha

Nos dois anos subsequentes à campanha, 6.970 pacientes foram submetidos a pelo menos uma medição adicional de creatinina. As características demográficas deste subgrupo permaneceram consistentes com as da população geral, com idade média de 55 ± 15 anos e 39% diagnosticados com DM.

As tendências da TFGe pós-campanha mostraram padrões distintos com base na função renal basal. Entre os pacientes com TFGe acima de 60 mL/min no momento da campanha, o declínio anual foi de 2,7 mL/min (IC 95%: 2,30–3,05). Por outro lado, aqueles com TFGe inferior a 60 mL/min apresentaram notável estabilidade durante o período de acompanhamento, com uma variação anual insignificante de -0,2 mL/min (IC 95%: -0,88 a 0,84).

### Risco de Insuficiência Renal

Também utilizamos a *Kidney Failure Risk Equation* (Equação de Risco de Insuficiência Renal) para estimar a probabilidade de insuficiência renal em 2 e 5 anos após o diagnóstico. O risco mediano para pacientes que preenchiam os critérios diagnósticos de DRC, seja com base na TFGe ou na RACU > 30 mg/g, foi de 4,1% em 2 anos e 15,8% em 5 anos. Na população de 1.630 pacientes para os quais estimamos o risco (todos os indivíduos com DRC na coorte), projetamos 14 pacientes evoluindo para insuficiência renal em 2 anos e 41 em 5 anos (Tabela 3).

## Discussão

Estudos de base populacional que estimam a prevalência de DRC continuam limitados em países de baixa e média renda. Em uma população de alto risco que participou de uma campanha nacional de rastreamento, constatamos que 1 em cada 5 pacientes com DM e/ou hipertensão arterial preenchia os critérios KDIGO para DRC. Nossos achados foram semelhantes aos dados relatados em uma grande coorte do NHANES, de 2007 a 2018, onde 17,5% dos adultos em risco de DRC foram identificados com albuminúria superior a 30 mg/g^13^. Embora as metodologias sejam diferentes, essa semelhança reforça o ônus da DRC não detectada em populações de alto risco.

De acordo com estimativas globais, aproximadamente 10% da população mundial apresenta DRC em seus diversos estágios. Em comparação com o ELSA, um estudo brasileiro prévio de base populacional, a prevalência aqui relatada para pacientes diabéticos e/ou hipertensos foi mais do que o dobro daquela observada na população adulta em geral^12^. No Brasil, a Sociedade Brasileira de Diabetes informa que existem cerca de 20 milhões de adultos com DM, e 60% deles desenvolverão DRC em algum momento. Nossas estimativas, utilizando uma equação validada, indicam uma projeção preocupante de aproximadamente 169.000 novos casos de insuficiência renal em pacientes diabéticos e hipertensos nos próximos 2 anos e 500.000 em 5 anos. Esses são dados alarmantes e estão em consonância com o que temos observado ao longo dos anos no censo da Sociedade Brasileira de Nefrologia. Nos últimos 15 anos, o número de pacientes em diálise duplicou, passando de 71.000 em 2006 para 154.000 em 2022^14^. Considerando as inovações que permitiram um melhor manejo dos fatores que contribuem para a mortalidade cardiovascular, o número estimado de pacientes subdiagnosticados e os achados deste estudo, as perspectivas para a saúde pública podem se agravar caso medidas de intervenção não sejam implementadas. É importante mencionar que essa estimativa não inclui casos de eventos cardiovasculares fatais ocorridos antes da insuficiência renal.

Outro achado importante do nosso estudo, além da elevada prevalência de DRC, foi a taxa extremamente baixa de testes de albuminúria, tanto antes como após a campanha. A albuminúria, uma medida fundamental para o diagnóstico e a estratificação de risco dos pacientes com a doença, é frequentemente negligenciada na prática clínica, não apenas nos países em desenvolvimento, mas também em nações desenvolvidas. Nossa expectativa era que, após a campanha, houvesse um aumento significativo na solicitação da RACU, especialmente na população em que identificamos albuminúria nos estágios A2 ou A3 de acordo com os critérios do KDIGO. Para nossa surpresa, menos de 9% dos pacientes com albuminúria A2 ou A3 identificados durante a campanha realizaram uma nova medição da RACU quando um teste de creatinina foi solicitado.

O presente estudo confirma nossos resultados anteriores publicados no estudo *Check CKD*, com base em mais de 4,5 milhões de testes de creatinina solicitados entre 2018 e 2021^8^. No *Check CKD*, o número de testes de albuminúria realizados juntamente com a creatinina foi maior, mas isso incluiu pacientes que já estavam sendo monitorados e cientes do seu diagnóstico de DRC. Outros estudos demonstraram uma taxa de testagem de 29% na França e 21% nos Estados Unidos em um subgrupo de pacientes em que a albuminúria deveria ter sido medida em todos os casos^15,16^. No estudo de Qiao et al.^17^, em um subgrupo de 35.000 pacientes sem inibidores do SRAA em suas prescrições, indivíduos com albuminúria A2 e A3 observaram um aumento na prescrição dessa classe de medicamentos de 37% e 43%, respectivamente. O estudo reforça que a medição da albuminúria tem sido associada ao aumento da prescrição de iSRAA^17^. A identificação da DRC por meio dos níveis de TFGe, complementada pela albuminúria, leva os médicos a iniciar ou intensificar terapias nefroprotetoras, aprimorando a adesão ao cuidado baseado nas diretrizes. Em nosso estudo, entretanto, menos de 10% dos indivíduos que realizaram o exame de creatinina sérica foram também submetidos ao teste de albuminúria, mesmo após receberem os resultados da campanha.

Campanhas de conscientização para pacientes com DRC, como a apresentada neste estudo, são essenciais para aprimorar a detecção precoce, intervenções oportunas, encaminhamento adequado a nefrologistas e manejo eficaz da doença. Além dos benefícios individuais, a conscientização do paciente pode desempenhar um papel fundamental na tomada de decisões compartilhadas e promover uma comunicação mais eficaz entre pacientes e profissionais de saúde. Essas ações podem desacelerar a progressão da doença, reduzir complicações, melhorar a qualidade de vida geral e diminuir o ônus econômico da DRC.

A inclinação da TFGe fornece uma medida robusta e sensível do declínio da função renal ao longo do tempo, oferecendo vantagens significativas tanto para a pesquisa quanto para aplicações clínicas. Em estudos observacionais, a inclinação da TFGe permite a avaliação da progressão da doença e a identificação de fatores de risco em ambientes de mundo real. Entretanto, em ECRs, serve como um desfecho substituto que pode detectar os efeitos do tratamento de forma mais precoce e precisa do que os desfechos tradicionais, como a progressão para doença renal em estágio terminal ou mortalidade. Por esse motivo, também exploramos esses dados durante os períodos anteriores e posteriores à campanha. Observamos que os pacientes com algum grau de DRC, seja albuminúria ou redução da TFGe, apresentaram uma progressão mais rápida do que seria tipicamente esperado com o envelhecimento normal no ano que antecedeu a campanha. O declínio anual de aproximadamente 1 mL/min por ano no grupo sem doença renal, classificado como verde pelo KDIGO, ressalta a qualidade dos dados apresentados, apesar da ausência de informações críticas sobre o manejo dos fatores de risco para a progressão da doença renal (Figura 3). Um achado interessante, apesar das limitações inerentes ao desenho do estudo, foi a redução na taxa de declínio da TFGe nos três subgrupos com doença renal crônica (Figura 4). Embora apenas uma pequena porcentagem dos pacientes tenha tido a albuminúria medida nos dois anos seguintes ao diagnóstico, esse fato pode, indiretamente, destacar um dos potenciais benefícios clínicos das campanhas de rastreamento. Pacientes com TFGe <60 mL/min são mais propensos a serem reconhecidos como portadores de DRC e, portanto, receberem tratamento adequado de acordo com a GDMT. Em contrapartida, aqueles com fatores de risco, mas com TFGe preservada (≥60 mL/min), apresentam menor probabilidade de serem manejados como indivíduos de alto risco, o que pode explicar o risco persistente observado nesse subgrupo. No entanto, nosso estudo não foi desenhado para capturar esse mecanismo, e isso permanece uma interpretação especulativa.

**Figura 3:** TFGe média estratificada pelo risco de progressão segundo o KDIGO.

Notas – Inclinação anual estimada antes da campanha: Sem DRC (Verde) –1,39 (–0,90; –1,88); Risco baixo (Amarelo): –1,88 (–0,77; –2,99); Risco médio (Laranja): –3,77 (–3,37; –8,96); Risco elevado (Vermelho): –6,16 (–8,96; –3,37). Inclinação anual estimada após a campanha: Sem DRC (Verde) –2,79 (–2,52; –3,06); Risco baixo (Amarelo): –0,58 (0,04; –1,19); Risco médio (Laranja): –0,24 (1,06; –1,54); Risco elevado (Vermelho): –2,58 (–0,69; –4,47).

**Figura 4:** Inclinação anual da TFGe antes e após a campanha.

Os pacientes que não preencheram os critérios para DRC durante a campanha apresentaram um declínio mais lento na TFGe em comparação àqueles que o fizeram (dados não apresentados). Uma análise exploratória adicional de subgrupos-chave revelou que idosos, homens, indivíduos com DM ou obesidade apresentaram um declínio mais rápido na TFGe.

Por fim, em nossa coorte, pacientes idosos, aqueles com DM, sobrepeso ou obesidade e indivíduos do sexo masculino mostraram-se mais propensos a apresentar redução da TFGe e albuminúria durante a campanha de rastreamento. Por outro lado, pacientes hipertensos apresentaram maior risco de redução da TFGe, mas não de albuminúria. No entanto, é importante observar que nosso estudo incluiu apenas pacientes com pelo menos uma comorbidade, já que esse foi um critério de inclusão. Portanto, não se pode concluir se o risco de albuminúria teria sido elevado se o parâmetro de comparação fossem indivíduos não hipertensos e não diabéticos, uma vez que a referência foi necessariamente indivíduos com DM. Contudo, a prevalência de pacientes hipertensos com RAC > 30 mg/g em nosso estudo foi quatro vezes menor do que a relatada em um amplo estudo de coorte realizado em Portugal^13^.

Nosso estudo apresenta algumas limitações. Ele foi realizado principalmente em cidades da região Sudeste, o que pode limitar sua generalização. A participação foi voluntária, possivelmente favorecendo indivíduos com melhor acesso à saúde do que a população em geral, o que pode influenciar as estimativas de prevalência e os padrões de testagem. Os dados podem estar incompletos para participantes que realizaram exames fora da rede de laboratórios utilizados no estudo. Embora os testes tenham seguido procedimentos padronizados, diversos locais estiveram envolvidos. Apenas uma única medição de TFGe e de RACU foi utilizada, e raramente foi possível realizar testes confirmatórios dentro de 90 dias. O uso do método de *Jaffé* para medição da creatinina constitui outra limitação, uma vez que este ensaio está sujeito a interferências analíticas (por exemplo, glicose, cetonas, bilirrubina e determinados medicamentos), que podem enviesar ligeiramente os resultados, apesar da calibração padronizada. Por fim, o estudo não avaliou se o diagnóstico precoce de DRC resultou em melhores desfechos ou maior adesão ao tratamento.

Apesar dessas limitações, campanhas como a relatada neste estudo são essenciais para melhorar a detecção precoce da DRC, ampliar a conscientização pública e incentivar indivíduos de alto risco a buscar atendimento médico. No Brasil, os exames de creatinina sérica e a RACU (assim como iRAAS e iSGLT2) estão universalmente disponíveis pelo sistema público de saúde, sem barreiras formais para sua solicitação. No entanto, o subdiagnóstico, a estratificação de risco incompleta e a subutilização da GDMT persistem. Tais iniciativas desempenham um papel crucial no desenvolvimento de estratégias direcionadas para prevenir a progressão da DRC e reduzir seu ônus econômico e à saúde no longo prazo.

Em conclusão, descrevemos uma elevada prevalência de DRC entre indivíduos com DM e hipertensão arterial sem histórico da doença. Além disso, persistem desafios significativos na complementação da TFGe com o teste de albuminúria — o biomarcador mais precoce para a detecção da DRC — na prática clínica. As tendências observadas na inclinação da TFGe antes e após a campanha (que incluiu a divulgação dos resultados dos testes de rastreamento da DRC aos participantes) sugerem que a identificação e a estratificação de risco dos pacientes, juntamente com a conscientização sobre a DRC, podem influenciar os desfechos, potencialmente por meio de mudanças nos padrões de prática e melhorias no manejo. Essa observação merece uma investigação mais aprofundada, mas reforça a importância do rastreamento da DRC em populações de alto risco.

## Data Availability

Os conjuntos de dados gerados e/ou analisados durante o presente estudo estão disponíveis mediante solicitação razoável ao autor correspondente.
